# The Potential Benefit of a Novel Urine Biosensor Platform for Lung Cancer Detection in the Decision-Making Process: From the Bench to the Bedside

**DOI:** 10.3390/jcm13206164

**Published:** 2024-10-16

**Authors:** Ory Wiesel, Tatiyana Suharev, Alaa Awad, Lina Abzah, Adi Laser-Azogui, Michal Mark Danieli

**Affiliations:** 1 Baruch Padeh—Tzafon Medical Center, Affiliated with the Azrieli Faculty of Medicine, Bar-Ilan University, Zefad 1528001, Israel; owiesel@tzmc.gov.il (O.W.); alaa_awad9@hotmail.com (A.A.); labzah@tzmc.gov.il (L.A.); 2 EARLY O.M. Ltd., Netanya 4250363, Israel; lab@earlylabs.io (T.S.); adi@earlylabs.io (A.L.-A.)

**Keywords:** volatile organic compounds, early-detection, cancer-diagnostics, screening, biomarkers, pulmonary, machine-learning, lung cancer, biosensors, biotechnology

## Abstract

**Background:** Lung cancer is the leading cause of cancer-related mortality worldwide. Lung cancer screening and early detection resulted in a decrease in cancer-specific mortality; however, it introduced additional dilemmas and adherence barriers for patients and providers. **Methods:** Innovations such as biomolecular diagnosis and biosensor-based technology improve the detection and stratification of high-risk patients and might assist in overcoming adherence barriers, hence providing new horizons for better selection of screened populations. **Conclusions:** In the present manuscript, we discuss some of the dilemmas clinicians are currently facing during the diagnosis and treatment processes. We further highlight the potential benefits of a novel biosensor platform for lung cancer detection during the decision making process surrounding lung cancer.

## 1. Introduction

Lung cancer (LC) remains a leading cause of cancer-related mortality worldwide. Lung cancer screening (LCS) protocols, using low-dose computed tomography scans (LDCT) has been shown to reduce lung-cancer-specific mortality worldwide compared with chest radiography or no screening [1,2]. Unfortunately, the benefit of LCS has not yet been translated into optimal utilization of its potential, with only 5–12% of eligible individuals actually being screened [3,4,5]. Non-adherence is another identified barrier, supported by significant figures in real-world data [6,7]. To date, the optimal clinical effectiveness and cost effectiveness of LCS is yet to be determined, and population-based LCS is still not considered standard practice in most countries and societies.

Multiple risk models for the selection of eligible individuals for LCS have been developed, helping to improve the selection of eligible patients for screening [8,9]. Once screened, both clinicians and patients are facing complex decisions regarding follow-up, diagnosis, and treatments based on the screening results in order to prevent overdiagnosis and overtreatment [10,11].

Current lung cancer screening programs are based on the National Lung Cancer Screening Trial (NLST) using LDCT [2]. However, these tests are associated with overdiagnosis, disparities in adherence, low positive predictive values, as well as a high cumulative false positive rate when used sequentially [12]. Moreover, the long elapsing time between referral and test, the duration of the test itself, and the test’s inconvenience all contribute to the low test utilization within the eligible population [13]. The benefit of BSP is its simplicity, ease of use, repeatability, low cost, benign lesion agnostics, non-invasiveness, and high sensitivity to early case cancers.

Although multiple risk prediction models have been developed to define the risk of detecting pulmonary nodules, they lack external validation [14]. Different molecular technologies aiming to detect biomarkers in biofluids such as sputum, blood, serum, and urine, or in exhaled breath and bronchial mucosa, are constantly being investigated [15]. These modern technologies, together with imaging, radiomics, and artificial intelligence, aimed to refine the risk, improve the selection of individuals who are eligible for screening, and improve the characterization of undetermined nodules detected in LDCT scans [14]. 

There is an urgent need for developing a rapid, simple, and reliable lung cancer detection test [16] which will facilitate diagnosis by the physician, in combination with traditional imaging techniques, and potentially serve as an alternative screening test in the future. Novel biosensor-based screening approaches for the identification of populations at risk, or even the diagnosis of lung cancer, are likely to play a significant role in future diagnostic and therapeutic algorithms [17]. 

Volatile organic compounds (VOCs) have been widely investigated in connection with cancer detection [18,19,20,21,22,23,24]. VOC levels can reflect pathophysiological processes including inflammation, necrosis, and cancer development, and various VOC profiles have been associated with lung cancer [25,26,27,28].

Although the detection of lung and other cancers in the breath and urine of patients by sniffer dogs has been reported several times [29,30,31,32,33,34], the actual compounds that are responsible for the scent are largely unknown. 

Due to their volatility at ambient temperatures, VOCs produced within cancer tissues travel in the systemic circulation before being freely excreted. In humans, VOCs have been detected in a wide range of samples, including breath, urine, blood, feces, tissue, and skin [35].

Compared to other biological samples, urine has the advantage of being easy and inexpensive to collect and handle. The selection of urine over breath as a non-invasive matrix should provide considerably more protection against adverse effects during sampling and analysis. The most important advantage of urine over breath is that urine, as a liquid phase, allows for its dispensing and partitioning into numerous subsamples, which is a considerable advantage over breath and a basic necessity for conducting appropriate quality control measures, and it may be stored for a nearly unlimited amount of time after freezing [35].

Recently, we have described a detection system that utilizes the extremely sensitive abilities of rats to detect VOCs from urine samples of patients to provide an indication of lung cancer [36]. Using the biosensor platform (BSP) in a double-blind study, we validated the presence of lung-cancer-specific VOC signatures in a single urine sample with 93% sensitivity and 91% specificity, suggesting its potential benefit for lung cancer detection and screening [36].

As there is a constant need for external validation and stratification tools during the process of pulmonary nodule detection, the goal of the current study is to discuss some of the obstacles clinicians are currently facing during the process of lung cancer diagnosis and to further analyze the potential benefit of this novel urine biosensor platform during the diagnostic pathway of complex patients with suspicious pulmonary nodules. 

## 2. Methods

The biosensor platform (BSP) for urine tests utilizes animals’ sensitive scenting abilities to detect lung cancer VOCs [37]. The platform combines biosensors (BSs), mechanical components, hardware, software, and a cloud-based database infrastructure. 

### 2.1. Biosensor Characteristics and Qualification

Long-Evans rats were inbred and raised in the Early Labs facility. The company prioritizes animal welfare, adhering to the strictest Welfare Quality^®^ standards set by the American Veterinary Medical Association (AVMA) [38]. To be included in the study, animals had to be clinically healthy and familiar with training and testing of odor discrimination procedures.

A multiphase protocol was developed for training the BSs using an automatic platform. 

The protocol comprises basic training, BSP sessions (designed to familiarize the BS with the technological environment), binary sessions, and repeated internal training. The training protocol is based on the behavioral shape methodology and includes several consecutive steps of basic training, associated learning, and detection reporting. The BSs learn to differentiate between urine samples of an LC origin versus those who do not bear LC and report a binary answer for a sample. At each stage, there is a mandatory threshold beyond which the next stage is initiated. Each training session contains between 35 and 60 separate and consecutive exposures to different urine samples in a predefined sequence. Once the animal has passed all training stages, it can move to the final test that is divided into two phases. The first includes testing samples that were used throughout the training sessions. In the second phase, the rat is exposed to a sequence of unknown samples in a double-blind test design: the test is conducted while the animal and the BSP operator are both blinded to the result.

### 2.2. The Biosensor Platform (BSP)

The system comprises two principal components: the “operational area”, where the controller and human operator are present, and the sample tube holders, which are loaded onto a conveyor belt. The second component is the “testing area”, where three identical smart pods for three independent BSs are located. Each BS operates separately within an isolated smart pod to ensure that they cannot influence one another. The samples are conveyed to the BS’s pods, and when a sample holder reaches a hole attached to the exact front location of a sniffing hatch, it opens, and the BS sniffs and reports its answer. Up to three samples can be assessed simultaneously by the platform, and each sample is tested by several separate and independent BSs. After the sample has been tested, the ventilation system is turned on to remove any remaining VOC residue, and the sample is moved to the next smart pod for another BS testing. Detailed specifications and characterization of the BSP, as well as figures, tables, and metrics, are thoroughly described in our previous publication [36].

### 2.3. BS Performance Analysis

The pods contain different sensors, recording the BS’s response to the urine odor, and the readings are passed as parameters to a machine learning algorithm. The duration of stay of the animal in the sniffing shaft is monitored by a sensor connected to a timer that counts the number of seconds in which the animal’s head is inside the sniffing hatch. Performance parameters, including sensitivity and specificity, are measured and analyzed during all stages of BS training. All data regarding every BS-SAMPLE interaction are automatically reported to the software. An algorithm, based on a unique model which quantifies different animal behavioral aspects, yields a highly accurate cancer risk assessment output. 

### 2.4. Urine Sample Preparation

Following collection in a sterile container by the patient, urine samples were aliquoted into 10 mL portions and stored in sterile polypropylene vacuum tubes at −20 °C. The preparation of the samples for the tests was performed under a hood; the urine was aliquoted into 0.3 mL samples, inserted into sample tube holders (to keep the sample isolated from the environment), and preheated to 60 °C before being placed onto the platform for testing.

### 2.5. Clinical Data

The following 5 representative clinical cases were selected to highlight the potential benefit of the novel BSP for lung cancer detection during the diagnosis and treatment of lung cancer. All patients were high-risk surgical candidates where a clinical dilemma was presented to our multidisciplinary team (MDT) during the diagnosis process.

All patients were enrolled in the clinical study prior to their definite diagnosis and signed an informed consent form during enrollment. At this point, as the BSP is not validated as a gold standard, none of our clinical decisions nor treatment protocols were influenced by the study results. The clinicians were blinded to the BSP test results during the process of diagnosis, MDT discussion, and treatments. The results of the BSP were only retrieved retrospectively during the preparation of this manuscript. This study was approved by IRB (# 0051-22-POR).

## 3. Results

### 3.1. The Patient That Cannot Undergo Biopsy

**Figure 1:**
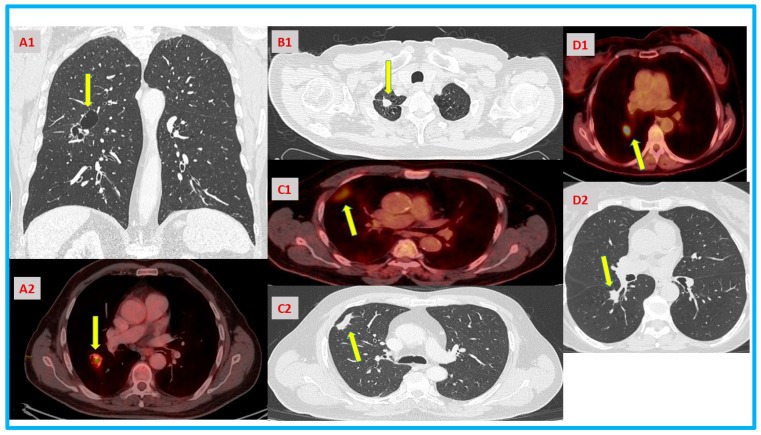
(**A**–**D**) Computed tomography and PET-CT scans of the presented patients. (**A1**) The arrow marks the suspicious cavitary lesion in the major fissure extending from the RLL to the RUL. (**A2**) The arrow marks the central hypermetabolic FDG avid nodule in the RLL. (**B1**) The arrow marks the apical mass in the vicinity of the emphysematous lung. (**C1**) The arrow marks the peripheral consolidation (on biopsy: chronic inflammation). (**C2**) The arrow marks the peripheral hypermetabolic FDG avid consolidation (on biopsy: chronic inflammation). (**D1**) The arrow marks a central hypermetabolic FDG avid nodule near the segmental pulmonary artery (biopsy was negative for tumor). (**D2**) The arrow marks central nodule (biopsy was negative for tumor).

Patient A: A 78-year-old male was referred for evaluation due to severe reflux. His past medical history was significant for lung infection of his right lung, complicated by lung abscess 40 years ago. He underwent a computed tomography scan and was found to have a medium-sized hiatal hernia and a cavitation with a 1.5 cm solid component in the right major fissure abutting both the right upper and right lower lobes. Pulmonary function tests showed a mild obstructive pattern. His Positron emission tomography scan (PET-FDG) showed standardized uptake values (SUVs) of 6.5 in the solid component of the cavitation (Figure 1, image A1,2). A consultation by an interventional radiologist concluded that given its deep location in the major fissure, the cavitation was not amenable for a needle biopsy nor for a navigation-based biopsy. A bronchoscopy with Broncho-alveolar-lavage yielded atypical cells from the right lower lobe basilar segment bronchi. The brain MRI did not show metastatic disease. Following a multidisciplinary team discussion, he was sent for thoracic surgery evaluation for resection. Given the central location of the lesion and the involvement of both the right upper lobe and right lower lobe, a major pulmonary resection was discussed with the patient (lower lobectomy with posterior upper lobe segmentectomy, and even pneumonectomy). The patient was hesitant to undergo surgery without a clear oncological tissue diagnosis given his age and chronic obstructive pulmonary disease (COPD). However, following multiple discussions with him and his family, he underwent a right lower lobectomy with en-block of the right upper lobe posterior segmentectomy and mediastinal lymph node dissection. The final pathology showed Stage IIA squamous cell carcinoma.

The preoperative urine biosensor platform resulted in a positive output in the lung cancer risk assessment. 

Patient B: A 64-year-old male, past smoker, was referred for thoracic surgery evaluation. His past medical history was significant for cerebral hemorrhage due to aneurysm, hypothyroidism, severe COPD, ischemic heart disease, chronic renal failure, and secondary hyperparathyroidism. The CT scan showed a 32 mm right upper lobe apical mass in the vicinity of the bullous lung parenchyma and vascular structures (Figure 1, image B1). The PET CT demonstrated a mild FDG avid RUL mass without distant uptake. The endobronchial ultrasound (EBUS) was negative for malignant cells in the mediastinal nodes. The brain imaging did not show metastatic disease. Given the lesion size, the multidisciplinary team discussion favored upfront surgical resection over stereotactic radiation therapy. The patient and his family refused surgery without prior diagnosis of cancer due to his COPD and co-morbidities and elected stereotactic body radiation therapy (SBRT). 

The preoperative urine biosensor platform resulted in a highly positive output in the lung cancer risk assessment. 

### 3.2. The High-Risk Patient with a Prior Negative Lung Biopsy

Patient C: A 64-year-old male, heavy smoker (100 pack-years), with past medical history of severe COPD, ischemic heart disease (status: post-PTCA and four coronary stents) was referred for evaluation following a computed tomography scan which demonstrated 1.8 cm right upper lobe consolidation without a solid component (Figure 1, image C1,2). The patient was treated with antibiotics by his pulmonologist; however, the consolidation did not resolve in a follow-up scan 2 months later. The patient was sent for a PET-CT, which showed mild FDG avidity (SUV 3.2) in the consolidation without other abnormalities. The CT-guided fine-needle biopsy indicated chronic inflammation, which was noted in the biopsy result without tumor cells. Another scan 3 months later showed slight enlargement of the consolidation, which prompted us to recommend surgical resection for a definitive diagnosis. A thoracoscopic wedge resection with adequate margins with lymph node sampling was performed, and a diagnosis of stage IB squamous cell carcinoma was established.

The preoperative urine biosensor platform resulted in a highly positive output in the lung cancer risk assessment. 

Patient D: A 68-year-old female, a current smoker with 120 pack-years was referred for evaluation to our pulmonary nodule clinic and was found to have a growing 1.8 cm solid nodule at the right lower lobe’s superior segment at the top of the bronchus (Figure 1, image D1,2). The nodule had significant FDG uptake. The patient underwent a bronchoscopic evaluation together with endobronchial ultrasound biopsies and navigation bronchoscopy with biopsies, and all were negative for malignancy. Her forced expiratory volume at 1 s was 1.35 L (56% predicted), her functional residual capacity was 2.2 L (73% of predicted), and her diffusion level of carbon monoxide was 50%. She barely passed her 6 min walk test. VO2 max was 15 mL/(kg*min). Given her borderline pulmonary status, she was hesitant to undergo upfront resection without a definite diagnosis. The patient was sent for SBRT. 

The preoperative urine biosensor platform resulted in a highly positive output in the lung cancer risk assessment. 

Patient E: A 58-year-old male with significant past medical history of morbid obesity (BMI 40), diabetes mellitus, ischemic heart disease, hypertension, and chronic lymphocytic leukemia. The patient was referred to our pulmonary nodule clinic for evaluation of a new 20 mm right upper lobe solid pulmonary nodule, depicted in a routine follow-up computed tomography scan. A meticulous review of his scan 12 months prior revealed tiny sub-centimeter pulmonary nodules in the right middle and lower lobes, as well as the left upper lobe, which were unchanged in the current scan. The PET-CT scan showed mild uptake in the new right upper lobe (SUV 2.5). Given his previous CLL, a computed-tomography-guided fine-needle biopsy yielded nondiagnostic results with no cancer cells. Another biopsy was performed, and the patient was diagnosed with squamous cell carcinoma. The patient underwent sub-lobar resection with mediastinal lymph node dissection. The pathology revealed stage IB squamous cell carcinoma.

The preoperative urine biosensor platform resulted in a highly positive output in the lung cancer risk assessment.

## 4. Discussion

Lung cancer is the leading cause of cancer-related mortality worldwide. In 2023, an estimated 238,340 new cases of lung cancer were diagnosed in the USA, with 127,070 deaths [5]. The majority of patients are diagnosed in the advanced stages, which decreases the chance for efficient treatment and long-term survival. 

Facilitating early detection and prompt treatment, cancer screening has been proven to decrease the rate of mortality in other solid organ cancers (colon, breast, cervix). The logic of screening for lung cancer prompted the National Lung Screening Trial (NLST), a phase 3 randomized trial, which demonstrated for the first time the benefit of screening high-risk populations using low-dose CT scans over chest radiographs [1]. In 2020, a 10-year follow-up of the NELSON trial further emphasized the importance of LCS by a reduction in lung cancer mortality in men by 24% (cumulative rate ratio for death from lung cancer: 0.76; 95% CI, 0.61–0.94; *p* = 0.01) [2]. Based on these large randomized trials, the NCCN, ACS, and US preventive services task force (SCPSTF), the American College of Chest Physicians, the European Society for Medical Oncology (ESMO), and other organizations world-wide implemented screening for high-risk individuals who are current or previous smokers [5,39,40,41,42]. 

Despite these strong recommendations, there is extreme variability in the utilization, adherence, and implementation of LCS protocols. Racial, ethnic, and gender disparities, especially in marginalized or rural communities, are some of the barriers for lung cancer screening [43,44]. These include not only patients but also providers and system-based disparities. Racial minorities and women at higher risk are more likely to be diagnosed with lung cancer at a younger age. They may have lower rates of smoking history, which leads to higher rates of ineligibility for lung cancer screening compared to older men and women. These disparities are examples of the challenges faced in addressing lung cancer.

As a result of the growing number of suspicious pulmonary nodules detected in LCS scans, there is a need to increase the thoracic surgery workforce to accommodate the amount of surgically operable cases [45]. On the other hand, given the relatively high false positive results that come with LCS, there is an emerging need to develop additional tools for better patient selection protocols for further work-up of detected nodules during LCS.

Appropriate risk stratification with risk prediction models might improve screening protocols to become more personalized with better efficacy and accuracy. Modern radiomics and imaging, together with molecular biomarkers detected in breath, blood, or urine samples (either by technology or by biosensor-based systems), can reduce the rate of false positive results from CT scans and might help establish individualized protocols for the detection and diagnosis of pulmonary nodules. Validation adjuncts might also help extend the screening interval for patients who are stratified as low-risk, hence reducing exposure, improving compliance, and reducing associated costs [46]. 

As approximately 320 high-risk smoking patients need to be screened annually for 3 years to prevent one death from lung cancer, and as the false positive rate of screening scans reach up to 96%, there is an urgent need for better utilization of multiple resources to overcome the modern barriers of LCS [47]. 

Risk prediction models employing a simple and inexpensive biomarker assay that is easy to obtain, collect, and transfer during a single office visit or even over the counter might help overcome some of these barriers and disparities and increase the utilization of LCS [44,48]. Future prospective studies using adjuncts for risk stratification and validation for LDCT should commence as more and more societies and organizations call for the implementation and utilization of individualized low-dose CT scans for lung cancer screening programs [49,50,51]. 

Biomarkers are the focus of intense research to overcome two major limitations during the process of lung cancer detection and diagnosis: the selection of high-risk individuals for lung cancer screening and the differentiation of benign and malignant pulmonary nodules detected in computed tomography scans. While numerous biomarkers have been studied so far, their clinical benefits in the process of pulmonary nodules malignancy risk assessment and lung cancer screening stratification remain to be defined. 

Autoantibodies against p53, cancer-associated gene protein (CAGE), New York esophageal squamous cell carcinoma 1 (NY-ESO-1), SRY-box transcription factor (SOX2), GBU4-5, ELAV-like protein 4 (HuD), and melanoma-associated antigen A4 (MAGE-A4) to assess pulmonary nodule malignancy risk are only a few examples of the available markers detected in the blood-test-based assays used in the early detection of lung cancer [52]. Exhaled breath condensate (EBC) is a source of the DNA fragments and volatile organic compounds used in the analysis of sputum samples of high-risk individuals with suspected findings in LCS.

Although not approved for clinical use, several pre-screening biomarker tests for lung cancer screening stratification are currently being thoroughly studied. Cell-free DNA tests (cfDNA)—a non-invasive assay—are utilized in a large multisite prospective validation study, which is an example of such a test [53,54]. miRNA-based tests aiming for gene expression regulators are another target for research [55,56]. Sputum-based tests (LuCEDs) have been developed to detect abnormal bronchial mucosal epithelial cells using novel imaging technology followed by a cytopathologist review [57].

Artificial intelligence (AI)-based technologies can improve the efficacy of screening by combining the benefits of machine learning with biomolecular assays. New data insights might be added and incorporated into a comprehensive multi-modal data source that computes all inputs into a statistical biopsy, offering a personalized approach for screening and early cancer detection [58]. 

The combination of machine learning and biosensor-based techniques which are rapid, cost-effective tools, is being studied intensely these days for early lung and breast cancer detection [59]. 

In the clinical cases presented, all patients had a significant risk of surgery. In each case, clinical dilemmas were presented to our multidisciplinary team during the diagnosis and treatment pathways. Patient A is an elderly male with an incidental finding of complex lung cavitation with a solid component that was suspicious for malignancy. Unfortunately, the location and nature of the lesion were not amenable for biopsy, and hence, they mandated significant pulmonary resection without prior histologic confirmation. Considering the innate wish of patients and families to avoid significant surgical interventions with potential major morbidity, adjunct validation studies might abate some of the confusion prior to shared decision making. The benefit of simple, non-invasive adjunct confirmatory test such as the urine biosensor platform might help during the preoperative discussion with potential surgical candidates. 

Patient B is an elderly male with significant co-morbidities, who presented with >3 cm lung mass in the right upper lobe. Given its location, in the vicinity of bullous disease, a needle biopsy was deemed a high risk. Both the patient and his family were reluctant to undergo surgery given his age and co-morbidities without histological evidence and postponed the decision and surgery. Although stereotactic radiation is a valid option for this group of patients, the benefit of SBRT for masses larger than 3 cm is unclear. With the risk of disease progression, the presence of confirmatory adjunct validation tests might assist with patient counseling and consent for surgery instead of SBRT and avoid unnecessary delays.

Patient C is an elderly high-risk heavy smoker who presented with a right upper lobe consolidation which did not improve in three consecutive scans. His PET-CT showed mild uptake at the lesion, and hence, a needle biopsy was carried out and yielded only chronic inflammatory cells. As repeated scans did not show clearance of the consolidation, the patient underwent surgical resection. His final pathology was significant for squamous cell carcinoma, which would have progressed otherwise. Adjunct confirmatory external validation of negative biopsy results in high-risk patients would prompt earlier interventions, such as surgical resection or repeated biopsy, which might help us avoid false diagnosis. This is another potential benefit of biomolecular studies such as the one presented. 

Similar to patient C, patient D is another high-risk heavy smoker with borderline–poor pulmonary function tests, presented after two separate negative biopsies of a highly suspicious growing central FDG avid nodule in the right lower lobe. SBRT was deemed a high risk given the proximity of the lesion to central segmental vessels and bronchi, and hence, a formal lobectomy was indicated given the location of the tumor. The presence of a positive adjunct external validation assay might help in the shared decision making weighing the risks and benefits of such a high-risk patient in electing the appropriate intervention among several high-risk interventions. 

Patient E is another example of a high-risk patient with morbid obesity with significant co-morbidities, who presented following a negative biopsy result of a growing right upper lobe among other stable multiple small bilateral lung nodules. The patient had a previous history of hematologic malignancy and significant co-morbidities, which prompted another needle biopsy, as a confirmation of the recurrence of his previous hematologic cancer would be a contraindication for surgical resection. Having a confirmatory adjunct test might avoid the risk of unnecessary additional invasive procedures (such as a second biopsy) and prompt a definitive surgical resection.

In all five cases provided, the benefit of adjunct non-invasive confirmatory test was presented. In the era of individualized medicine, additional cost-effective, non-invasive tests such as the presented BSP might save unnecessary invasive biopsies and costly tests, expedite diagnostic and treatment decisions in complex patients, assist in shared decision making, and—once validated—help in the follow-up of patients and early detection of recurrent or metastatic disease.

Clearly, each new test has its limitations. First and foremost, there is a need for further clinical validation of the BSP prior to any clinical decision making and commercial use. Proper clinical trials should be performed besides proof-of-concept studies. The use of additional tests might add to the delay in the diagnostic pathway, although a BSP-based protocol might be carried out in parallel and is meant to provide a rapid indication of whether the patient has lung cancer or not. While one of the major limitations of the test is its inability to indicate the sub-type and the stage of lung cancer, its use might strengthen the diagnosis and assist in treatment decisions, specifically for high-risk patients and their clinicians. Lastly, although it is not intended to replace proper tissue diagnosis, the balance between the test’s low cost compared to the current CT scan-based programs, the simplicity of performing the test in any place (physician’s office or at home) might help to improve the low adherence rates of patients and clinicians in LCS programs. 

## 5. Conclusions

The early detection of lung cancer has been proven to save lives. The utilization of innovative approaches such as the detection of biomolecules with the help of novel AI-based biosensor platforms might improve the adherence to the current screening protocols and assist in the diagnosis and decision making process of detected non-specific pulmonary nodules (Table 1, Table 2 and Table 3).

## Figures and Tables

**Table 1:** Potential utilization of the urine biosensor platform.

Identify pre-invasive lesions not identified on CT scans.
Pre-screen population to better utilize health care costs.
Confirm findings on LDCT and screening scans.
Identify populations at risk.
Identify suspicious nodules in endemic infection areas.
Post-operative follow-up and early diagnosis of recurrent disease.

**Table 2:** Potential benefits of the urine biosensor platform.

Problem	Barrier	Potential Benefit Using BSP	Comments
Fear of unnecessary radiation exposure	Adherence—patients	Easy to perform, non-invasive, cheap	Can be integrated in other screening protocols
Complex referral pathways	Adherence—physicians	Easy to perform, cheap	Can be integrated in other screening protocols
Poor countries or communities	Utilization of resources	Cheap, can be integrated with other tests	Area with limited resources for LCS programs
Special populations—sick, elderly	Decision making	Can help in complex clinical decisions and borderline patients	Alternative treatments, potential candidates for research protocols
Complex nodules to biopsy	Decision making	Can help in complex clinical decisions and borderline patients	Can define risk benefit for biopsies in high-risk patients
Endemic regions with pulmonary nodules	Diagnostic protocols	Define population for further work-up (biopsy, etc.)	
Post-resection or treatment for lung cancer	Diagnostic protocols	Easy to perform, cheep, non-invasive	Early detection of metastases or recurrence

**Table 3:** Clinical case summary with potential benefits of the BSP.

Case	What Is the Problem	Performed Procedures	How the BSP Could Change Management
A	Cavitation with solid component that is not amenable for biopsy would need large resection. Elderly high-risk patient with a limited pulmonary reserve.	Bronchoalveolar lavage.	Validated positive result would help with shared decision making and avoid delay prior to surgery.
B	>3 cm lung mass in high-risk patient, not amenable for biopsy.	Patient request to postpone surgery for additional imaging. Later agreed for SBRT.	Validated positive result might help with shared decision making and avoid delay for definitive treatment.
C	Persistent consolidation in high-risk patient with negative biopsy.	Needle biopsy, follow-up repeated scans at 3,6 months. Delayed surgery finally.	Validated positive result would prompt earlier surgical intervention. Might avoid stage migration and adjuvant chemotherapy.
D	Borderline pulmonary function tests for lobectomy in high-risk patient for both lobectomy and SBRT.	EBUS, navigation bronchoscopy. Offered surgical resection.	Validated positive result would prompt earlier surgical resection. Would help in discussing the risks vs. benefits of surgery vs. SBRT with the patient.
E	Enlarging pulmonary nodule in previous hematologic cancer patient with additional tiny nodules.	Needle biopsy ×2.	Validated positive result would avoid second biopsy and prompt earlier surgical resection for definitive treatment.

## Data Availability

The original contributions presented in the study are included in the article, further inquiries can be directed to the corresponding author.
